# Association of Recreational Cannabis Legalization With Cannabis Possession Arrest Rates in the US

**DOI:** 10.1001/jamanetworkopen.2022.44922

**Published:** 2022-12-05

**Authors:** Christian Gunadi, Yuyan Shi

**Affiliations:** 1 Herbert Wertheim School of Public Health and Human Longevity Science, University of California San Diego, La Jolla

## Abstract

**Importance:**

Recreational cannabis legalization (RCL) has been advocated as a way to reduce the number of individuals interacting with the US criminal justice system; in theory, however, cannabis decriminalization can achieve this objective without generating the negative public health consequences associated with RCL. It is still unclear whether RCL can bring additional benefits in terms of reducing cannabis possession arrests in states that have already decriminalized cannabis.

**Objective:**

To examine whether RCL was associated with changes in cannabis possession arrests in US states that had already decriminalized cannabis during the study period and whether these changes differed across age and racial subgroups.

**Design, Setting, and Participants:**

This repeated cross-sectional study used cannabis possession arrest data from the Federal Bureau of Investigation Uniform Crime Reporting Program (UCRP) for US states from 2010 through 2019. Statistical analysis was conducted from October 6, 2021, to October 12, 2022.

**Exposures:**

Implementation of statewide RCL.

**Main Outcomes and Measures:**

Cannabis possession arrest rates per 1000 population per year were assessed with a quasi-experimental difference-in-differences design and were used to estimate the association of RCL with arrest rates in RCL states that had or had not decriminalized cannabis before RCL. This association was also examined in subgroups for age (adults vs youths) and race (Black vs White).

**Results:**

This study included UCRP data for 31 US states, including 9 states that implemented RCL during the study period (4 without and 5 with decriminalization) and 22 non-RCL states. In the 4 states that had not decriminalized cannabis before legalization, RCL was associated with a 76.3% decrease (95% CI, −81.2% to −69.9%) in arrest rates among adults. In the 5 states that had already decriminalized cannabis, RCL was still associated with a substantial decrease in adult arrest rates (−40.0%; 95% CI, −55.1% to −19.8%). There was no association of RCL with changes in arrest rates among youths. In addition, changes in arrest rates associated with RCL did not differ among Black and White individuals.

**Conclusions and Relevance:**

In this repeated cross-sectional study, RCL was associated with a sizable reduction in cannabis possession arrests among adults in states that had already decriminalized cannabis during the study period (2010-2019), albeit the magnitude was smaller compared with states that had not decriminalized cannabis before RCL. In addition, RCL did not seem to be associated with changes in arrest rates among youths or disparities in arrest rates among Black and White individuals.

## Key Points

**Question:**

Is recreational cannabis legalization (RCL) associated with a reduction in cannabis possession arrest rates in US states that have already decriminalized cannabis; if so, does this change differ by age and race?

**Findings:**

In this cross-sectional study using difference-in-differences analysis for data from 31 states, RCL was associated with a 40% reduction in cannabis possession arrest rates among adults in the 5 states that had already decriminalized cannabis between 2010 and 2019. This decrease was significantly smaller than that for the 4 states that had not decriminalized cannabis (76%), and there was no association between RCL and changes in youth arrest rates or disparities in arrest rates among Black and White individuals.

**Meaning:**

These findings suggest that RCL was associated with decreased cannabis possession arrest rates among adults during the study period, even in US states that had already decriminalized cannabis.

## Teaser

This cross-sectional study examines whether recreational cannabis legalization was associated with changes in cannabis possession arrest rates in the US between 2010 and 2019, including by state decriminalization status and by age and race.

##  Introduction

In 2012, Colorado and Washington became the first US states to implement recreational cannabis legalization (RCL), which allows the possession of a small amount of cannabis for adult use without any penalties. Since then, 16 states and the District of Columbia have followed suit.^1^ One reason for this momentum is the increasing recognition that cannabis illegality sends a large number of individuals to the criminal justice system, with adverse physical, mental, and social consequences.^2^

To reduce the number of cannabis possession arrests, civil liberties advocates have voiced support for RCL.^2^ In theory, however, a reduction in cannabis possession arrests can be achieved by decriminalizing cannabis, which changes the penalties associated with a small amount of cannabis possession from criminal to civil infractions. In fact, more than 30 states have adopted cannabis decriminalization since the 1970s. The latest research estimates that during the 2010s, cannabis possession arrest rates were reduced substantially among both adults and youths (by 40%-80%) as a result of decriminalization.^3,4,5^ Cannabis decriminalization and RCL have substantial differences in terms of motivations, enactment, provisions, and enforcements. Both, however, have the potential to reduce cannabis possession arrests. If the policy goal is solely to reduce arrests, decriminalization may seem to be more appealing than RCL. First, under cannabis decriminalization, individuals caught possessing a small amount of cannabis are still subject to civil penalties (eg, fines, mandated drug education programs, or public services) but do not receive criminal sanctions. The civil penalties may serve as a deterrent to cannabis use and prevent related adverse consequences. Second, RCL could allow the legal sale of cannabis, which increases access and exposure to cannabis products and marketing activities. The legality of cannabis may also make social norms more favorable to cannabis. Some studies have shown that RCL is associated with increased use of cannabis among both adults and youths, creating considerable public health concerns.^6,7,8,9^

In contrast, RCL has potential to further reduce cannabis possession arrests on top of decriminalization. Under RCL, law enforcement agents are no longer required to look for cannabis possession violations in a small amount. Although possession of a large amount of cannabis is still a criminal offense under both decriminalization and RCL, an overall lower police search rate under RCL will likely lead to a large reduction in arrests even in states that have already decriminalized cannabis.

We are aware of only 1 study that has examined the association of RCL with cannabis possession arrests.^5^ In a comparison of 4 US states that implemented RCL from 2000 to 2016 with other states that did not change penalties for cannabis possession in the same period, Plunk et al^5^ found that RCL was associated with a substantial decline in arrest rates among adults but not youths. The authors did not distinguish RCL states that had already decriminalized cannabis from those that had not.^5^ It remains unclear whether RCL can further reduce cannabis possession arrests in states that have already decriminalized cannabis.

This study had 2 objectives. First, we examined whether RCL implementation was associated with a reduction in cannabis possession arrests in states that had already decriminalized cannabis. If no evidence was found, it might be plausible for policy makers in states already implementing decriminalization to consider alternative strategies other than RCL to further reduce arrests. We also examined whether the association existed in states without cannabis decriminalization. If no evidence was found, decriminalization might be considered a more effective and safer strategy of reducing arrests without the potential negative consequences associated with RCL.

Second, we assessed whether the association of RCL with cannabis possession arrests differed by age (adults vs youths) and race (Black vs White). Recreational cannabis has been legalized only for adult use, which in theory should have minimal effects on arrests of youths. According to a previous American Civil Liberties Union report, Black individuals were considerably more likely to be arrested for cannabis possession compared with White individuals, despite a similar rate of cannabis use.^10^ The magnitude of the change in arrests after RCL could therefore differ among Black and White individuals.

## Methods

This cross-sectional study used publicly available secondary data sources and was therefore deemed exempt from institutional review board approval and informed consent per University of California San Diego policy. The study followed the Strengthening the Reporting of Observational Studies in Epidemiology (STROBE) reporting guideline.

### Primary Data Source

Data on cannabis possession arrests by age, sex, and race were obtained from the Uniform Crime Reporting Program (UCRP) for 2010 to 2019.^11^ The UCRP is assembled from more than 18 000 law enforcement agencies and maintained by the US Federal Bureau of Investigation, and it is one of the most widely used data sources to examine crimes in the US.^4,5,12^

UCRP arrest data have limitations. First, reporting to the UCRP by law enforcement agencies is voluntary; hence, some states in some periods may have incomplete data. In particular, Florida did not report arrest statistics for most years (2010-2016) in the study period; we thus excluded Florida from this study. Second, there was a misreporting in the number of arrests by the Denver Police Department after cannabis was legalized in Colorado.^5^ Because this department is one of the largest police divisions in Colorado, misreporting in Denver could result in incorrect statistics for the entire state; we consequently excluded Colorado from this study. Third, information on arrests by race and ethnicity was limited. Race information was available only for American Indian or Alaska Native, Asian, Black, and White individuals. Data on American Indian or Alaska Native and Asian individuals were not assessed in this study because cannabis possession arrests among these groups are rare and the disparity in arrest rates is most striking between Black and White individuals. Ethnicity information, such as Hispanic origin, was unavailable for most of the study period. Race and ethnicity information is often reported by officers and subject to inaccuracy. Finally, the UCRP adheres to the hierarchy rule in its reporting, such that less severe crimes (part II offenses) like cannabis possession are reported only if they occur during an incident without the more severe crimes (part I offenses).^13^

### Outcome: Cannabis Possession Arrest Rate

Our main outcome was annual cannabis possession arrest rates per 1000 population at the state level. The state-level population estimates were obtained from the Integrated Public Use Microdata Series American Community Survey (ACS) for 2010 to 2019.^14^ The rates were calculated separately in adult and youth populations and separately for Black and White individuals. Although the legal purchase age for recreational cannabis was 21 years in all RCL states, we used age 18 years as the cutoff to define adults and youths in this study because the UCRP reports arrests by race in only 2 age categories based on this cutoff.^11^ Eighteen years was also used as the cutoff age in previous literature.^4,5,12^

### Policy Exposure: RCL

The state-level policy exposure was a binary indicator taking the value of 1 if RCL was in effect in a year and a state and 0 otherwise.

There were 9 states that implemented RCL in the study period (eTable 1 in Supplement 1). Two groups of RCL states were constructed as follows based on whether cannabis decriminalization was already in place before RCL implementation^4,5,15,16,17^: (1) RCL states without decriminalization already in place, including Washington, Alaska, Nevada, and Michigan; and (2) RCL states with decriminalization already in place, including Oregon, California, Massachusetts, Maine, and Vermont. Because California and Vermont decriminalized cannabis during the study period, we excluded their state–year observations before and in the year of decriminalization. These 2 groups of RCL states were analyzed separately to allow differential effects of RCL by decriminalization status.

The comparison group consisted of 22 states that neither implemented RCL nor changed penalties for cannabis possession in the study period (ie, non-RCL states).^4,5,15,16,17^ Seventeen states that did not implement RCL were excluded from the study because they either decriminalized cannabis or changed penalties for cannabis possession during the study period.^2,4,5^ eTable 1 in Supplement 1 lists all included and excluded states.

### Covariates

Following previous studies, time-varying state-level covariates that may confound the association of RCL with cannabis possession arrests were included in the regressions.^3,4,5^ These covariates included the share of the population with less than a high school diploma or equivalent, share of female individuals in the population, share of individuals in the population from racial and ethnic minority groups, share of youths in the population, number of police officers per 1000 population, unemployment rate, income per capita in 2019 thousand dollars, and poverty rate. These state-level characteristics were constructed from ACS and UCRP data on law enforcement officers killed and assaulted.^18^ The binary indicator for the presence of medical cannabis legalization was also included in the regressions, taking the value of 1 if it was in effect in a year and a state and 0 otherwise.^4,5^ The effective dates of medical cannabis legalization were obtained from ProCon.org.

### Statistical Analysis

The unit of analysis was state–year observations. To examine the association of RCL with cannabis possession arrest rates, we used the quasi-experimental difference-in-differences research design. Specifically, we used log-linear regression to model arrest rates as a function of RCL, adjusting for all covariates mentioned in the previous section, fixed effects for state and year, and state-specific time trends. State fixed effects (indicators of states) accounted for potential state-specific confounding factors that did not vary over time. Year fixed effects (indicators of years) accounted for secular trends in outcomes common to all states in the same year. State-specific time trends (linear trend variable for each state) accounted for state-specific confounding factors that varied linearly over time. Two types of RCL states (with and without cannabis decriminalization) were compared with comparison states separately. The analysis was also conducted separately for adults and youths and for Black and White individuals.

Vermont reported 0 cannabis possession arrests for Black adults in 2015 and 2019. To avoid dropping these state–year observations, we added a small constant (0.01) to all state–year observations for log transformation in all race-related analysis. Our results were not sensitive to this specification.

As noted earlier, the voluntary reporting to UCRP potentially exerts bias in the estimated association due to measurement error. Because this error was more severe in areas with a small population size, we followed previous studies to weight the regression by state population size averaged over the entire study period to minimize potential bias.^19,20^ Standard errors were clustered at the state level to account for possible serial correlation within a state.^21^

We formally tested differences in the effects of RCL among (1) RCL states with and without decriminalization already in place, (2) adults and youths, and (3) Black and White individuals. Specifically, we used seemingly unrelated estimation and tested the equality between regression coefficients in the comparison.

We also conducted 2 supplemental analyses. First, we conducted an event study by replacing the RCL indicator with a series of its leads and lags to examine whether the estimated association was driven by the difference in prelegalization trends between RCL and non-RCL states. Second, we conducted a leave-one-out exercise by dropping 1 RCL state from the regression at a time to examine whether the overall estimate was driven by a specific RCL state. *P* < .05 was considered statistically significant. Statistical analyses were performed with Stata, version 16 (StataCorp LLC).

## Results

### Descriptive Statistics

Table 1 reports outcome and covariate statistical results by state RCL and decriminalization status. eFigure 1 in Supplement 1 plots trends in cannabis possession arrest rates in RCL states during the study period. We observed a substantial decrease in arrest rates after RCL was implemented in states that had already decriminalized cannabis, although this decline seemed smaller compared with RCL states that had not decriminalized cannabis. The magnitude of the decline seemed to be similar between Black and White individuals regardless of state decriminalization status. The decrease in arrest rate seemed to be driven by adults but not youths (eFigures 2 and 3 in Supplement 1, respectively).

**Table 1.** Summary Statistics

Analysis	Non-RCL States (2010-2019)		RCL states (year before legalization)			
	Mean (SD)	Median (IQR)	Decriminalization not in place		Decriminalization in place	
			Mean (SD)	Median (IQR)	Mean (SD)	Median (IQR)
Outcome: cannabis possession arrest rates per 1000 population by subgroup						
Overall population	2.32 (1.50)	2.27 (1.60-2.78)	1.43 (0.44)	1.52 (1.11-1.75)	0.88 (1.01)	0.20 (0.17-1.84)
Black	7.88 (5.70)	6.54 (4.97-9.68)	4.60 (2.50)	3.85 (3.02-6.19)	2.25 (2.61)	1.10 (0.30-3.24)
White	1.92 (1.47)	1.82 (1.24-2.29)	1.29 (0.37)	1.40 (1.02-1.56)	0.90 (1.01)	0.24 (0.19-1.81)
Among adults	2.66 (1.91)	2.62 (1.82-3.19)	1.59 (0.57)	1.68 (1.15-2.02)	0.91 (1.08)	0.17 (0.11-2.00)
Black	9.90 (6.88)	8.35 (6.27-12.15)	5.49 (3.47)	4.68 (3.19-7.79)	3.14 (4.18)	1.21 (0.29-3.85)
White	2.12 (1.81)	2.03 (1.39-2.52)	1.35 (0.43)	1.47 (1.07-1.63)	0.90 (1.07)	0.16 (0.15-1.96)
Among youth	1.23 (0.69)	1.14 (0.82-1.56)	0.91 (0.21)	0.84 (0.77-1.06)	0.75 (0.75)	0.38 (0.33-1.14)
Black	3.93 (12.58)	2.30 (1.42-3.41)	2.20 (1.19)	2.02 (1.41-3.00)	1.04 (0.80)	0.83 (0.64-1.54)
White	1.25 (0.73)	1.15 (0.79-1.66)	1.06 (0.33)	0.99 (0.83-1.29)	0.86 (0.84)	0.59 (0.33-1.17)
Covariate						
Share of population with less than a high school diploma/equivalent	0.33 (0.03)	0.32 (0.30-0.35)	0.32 (0.02)	0.32 (0.30-0.33)	0.29 (0.04)	0.28 (0.26-0.30)
Share of female individuals in the population	0.51 (0.01)	0.51 (0.50-0.51)	0.50 (0.02)	0.50 (0.49-0.50)	0.51 (0.00)	0.51 (0.50-0.51)
Share of individuals in the population from racial and ethnic minority groups	0.24 (0.15)	0.22 (0.14-0.32)	0.28 (0.07)	0.27 (0.22-0.34)	0.17 (0.14)	0.15 (0.06-0.21)
Share of youths (aged <18 y) in the population	0.24 (0.02)	0.23 (0.22-0.24)	0.23 (0.01)	0.23 (0.22-0.24)	0.21 (0.02)	0.20 (0.19-0.22)
Officers per 1000 population	2.23 (0.54)	2.10 (1.86-2.46)	1.83 (0.33)	1.80 (1.55-2.11)	2.04 (0.31)	1.96 (1.84-2.01)
Unemployment rate	0.06 (0.02)	0.05 (0.04-0.07)	0.06 (0.02)	0.06 (0.05-0.07)	0.05 (0.02)	0.05 (0.04-0.06)
Income per capita in 2019 thousand dollars	29.83 (4.31)	29.12 (26.68-31.89)	32.27 (2.43)	32.04 (30.46-34.08)	33.89 (4.46)	33.43 (31.11-34.08)
Poverty rate	0.14 (0.03)	0.14 (0.11-0.17)	0.13 (0.01)	0.14 (0.13-0.14)	0.13 (0.02)	0.13 (0.11-0.15)

Abbreviation: RCL, recreational cannabis legalization.

### Main Analysis

Table 2 reports association estimation results from regressions (detailed results in eTables 2-7 in Supplement 1). Table 3 reports the test statistics for the differences in association coefficients among (1) RCL states with and without cannabis decriminalization, (2) adults and youths, and (3) Black and White individuals.

**Table 2.** Association of Recreational Cannabis Legalization and Cannabis Possession Arrest Rates^a^

RCL status	Natural log of cannabis possession arrest rate per 1000 population		
	All races	Black	White
**States without decriminalization already in place**			
Overall population			
Coefficient (SE) [95% CI]^b^	−1.16 (0.12) [−1.41 to −0.91]	−1.21 (0.15) [−1.52 to −0.89]	−1.09 (0.10) [−1.30 to −0.88]
Percentage change^c^	−68.65	−70.18	−66.38
*P* value	<.001	<.001	<.001
Adults (aged ≥18 y)			
Coefficient (SE) [95% CI]	−1.44 (0.12) [−1.67 to −1.20]	−1.51 (0.16) [−1.85 to −1.18]	−1.37 (0.09) [−1.56 to −1.18]
Percentage change	−76.31	−77.91	−74.59
*P* value	<.001	<.001	<.001
Youths (aged <18 y)			
Coefficient (SE) [95% CI]	−0.38 (0.22) [−0.85 to 0.078]	−0.24 (0.27) [−0.81 to 0.32]	−0.36 (0.20) [−0.77 to 0.047]
Percentage change	−31.61	−21.34	−30.23
*P* value	.10	.38	.08
**States with decriminalization already in place**			
Overall population			
Coefficient (SE) [95% CI]	−0.40 (0.14) [−0.68 to −0.12]	−0.37 (0.14) [−0.66 to −0.085]	−0.41 (0.13) [−0.67 to −0.15]
Percentage change	−32.97	−30.93	−33.63
*P* value	.007	.01	.003
Adults			
Coefficient (SE) [95% CI]	−0.51 (0.14) [−0.80 to −0.22]	−0.43 (0.18) [−0.81 to −0.057]	−0.53 (0.13) [−0.80 to −0.27]
Percentage change	−39.95	−34.95	−41.14
*P* value	.001	.03	<.001
Youths			
Coefficient (SE) [95% CI]	−0.19 (0.10) [−0.39 to 0.021]	−0.10 (0.07) [−0.23 to 0.034]	−0.18 (0.09) [−0.37 to 0.01]
Percentage change	−17.30	−9.52	−16.47
*P* value	.08	.14	.07

Abbreviation: RCL, recreational cannabis legalization.

^a^ All regressions also included controls for the presence of medical cannabis legalization, share of the population with less than a high school diploma or equivalent, share of female individuals in the population, share of individuals in the population from racial and ethnic minority groups, share of youths in the population, number of police officers per 1000 population, unemployment rate, income per capita in 2019 thousand dollars, poverty rate, state and year indicators, and state-specific time trends. All regressions were weighted by state population averaged over the study period (2010-2019). Detailed regression results are reported in eTables 2 to 7 in Supplement 1.

^b^ Standard errors were clustered at the state level.

^c^ Percent change was obtained from (*e*^c^^oeffi^^c^^ient^ − 1).

**Table 3.** Differences in Association Estimation by Decriminalization Status of RCL and Population Age and Race in US States^a^

Sample	*P* value, equality test^b^
**Same rates in states with vs without decriminalization before RCL**	
Overall population	
All	<.001
Black	<.001
White	<.001
Adults (aged ≥18 years)	
All	<.001
Black	<.001
White	<.001
Youths (aged <18 years)	
All	.32
Black	.51
White	.30
**Same rates among adults vs youths**	
RCL states without decriminalization	
All	<.001
Black	<.001
White	<.001
RCL states with decriminalization	
All	<.001
Black	.04
White	<.001
**Same rates among Black vs White individuals**	
RCL states without decriminalization	
All	.17
Adults	.05
Youths	.18
RCL states with decriminalization	
All	.40
Adults	.12
Youths	.27

Abbreviation: RCL, recreational cannabis legalization.

^a^ All regressions also included controls for the presence of medical cannabis legalization, share of the population with less than a high school diploma or equivalent, share of female individuals in the population, share of individuals in the population from racial and ethnic minority groups, share of youths in the population, number of police officers per 1000 population, unemployment rate, income per capita in 2019 thousand dollars, poverty rate, state and year indicators, and state-specific time trends. Standard errors were clustered at the state level. All regressions were weighted by state population averaged over the study period (2010-2019).

^b^ *P* values in equality tests were obtained by using seemingly unrelated estimation and testing the equality between coefficients.

#### Overall Population

Recreational cannabis legalization was associated with a 68.6% decline (95% CI, −75.6% to −59.8%) in arrest rates in RCL states that had not decriminalized cannabis before RCL and a 33.0% decline (95% CI, −49.3% to −11.3%) in RCL states that had already decriminalized cannabis. The association difference in the 2 types of RCL states was statistically significant (*P* < .001).

#### By Age

Among adults, RCL was associated with a 76.3% decline (95% CI, −81.2% to −69.9%) in arrest rates in RCL states that had not decriminalized cannabis before RCL and a 40.0% decline (95% CI, −55.1% to −19.8%) in RCL states that had already decriminalized cannabis. Among youths, RCL was not associated with a change in arrest rate.

#### By Race

In RCL states that had not decriminalized cannabis before legalization, RCL was associated with a 77.9% decline (95% CI, −84.3% to −69.3%) in arrest rates among Black adults and a 74.6% decline (95% CI, −79.0% to −69.3%) among White adults. In RCL states that had already decriminalized cannabis before legalization, RCL was associated with a 35.0% decline (95% CI, −55.5% to −5.5%) in arrest rates among Black adults and a 41.1% decline (95% CI, −55.1% to −23.7%) among White adults. However, there was no evidence that the decreases differed between Black and White adults in either type of RCL state. There was no association between RCL and arrest rate in Black or White youths.

### Supplemental Analysis

**Figure 1.**
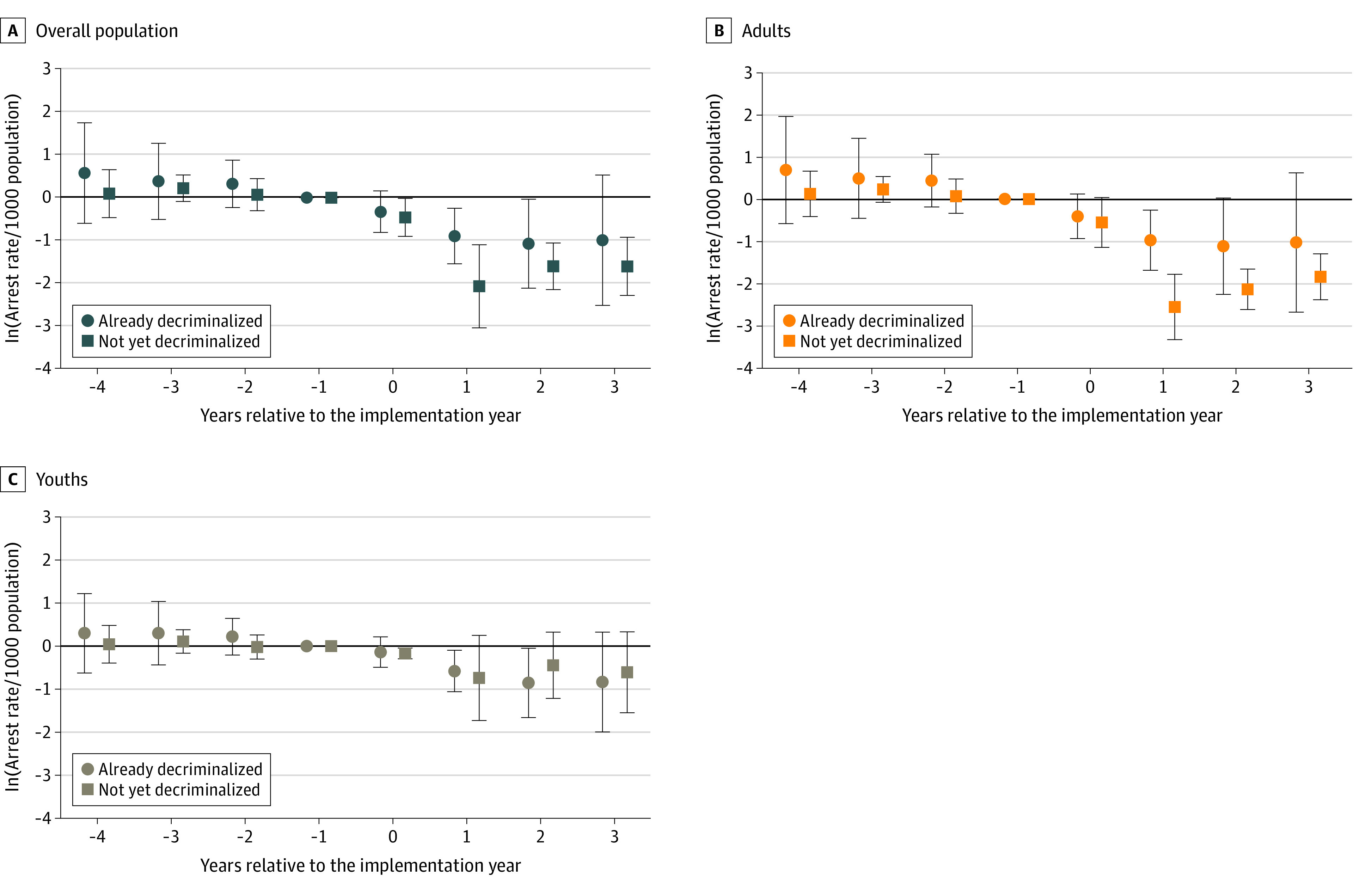
Event Study by Age Group, With Arrest Rates per 1000 Population per Year Estimated coefficients are shown for the overall population, adults, and youths; error bars indicate 95% CIs. The year before implementation was the reference (omitted) year. The estimated coefficient should be interpreted as relative to this year. The final lag or lead points accumulated all years beyond (ie, −4 included year −4 and earlier; 3 included year 3 and later). All regressions also included controls for the presence of medical cannabis legalization, share of the population with less than a high school diploma or equivalent, share of female individuals in the population, share of individuals in the population from racial and ethnic minority groups, share of youths in the population, number of police officers per 1000 population, unemployment rate, income per capita in 2019 thousand dollars, poverty rate, state and year indicators, and state-specific time trends. Standard errors were clustered at the state level. All regressions were weighted by state population averaged over the study period (2010-2019).

**Figure 2.**
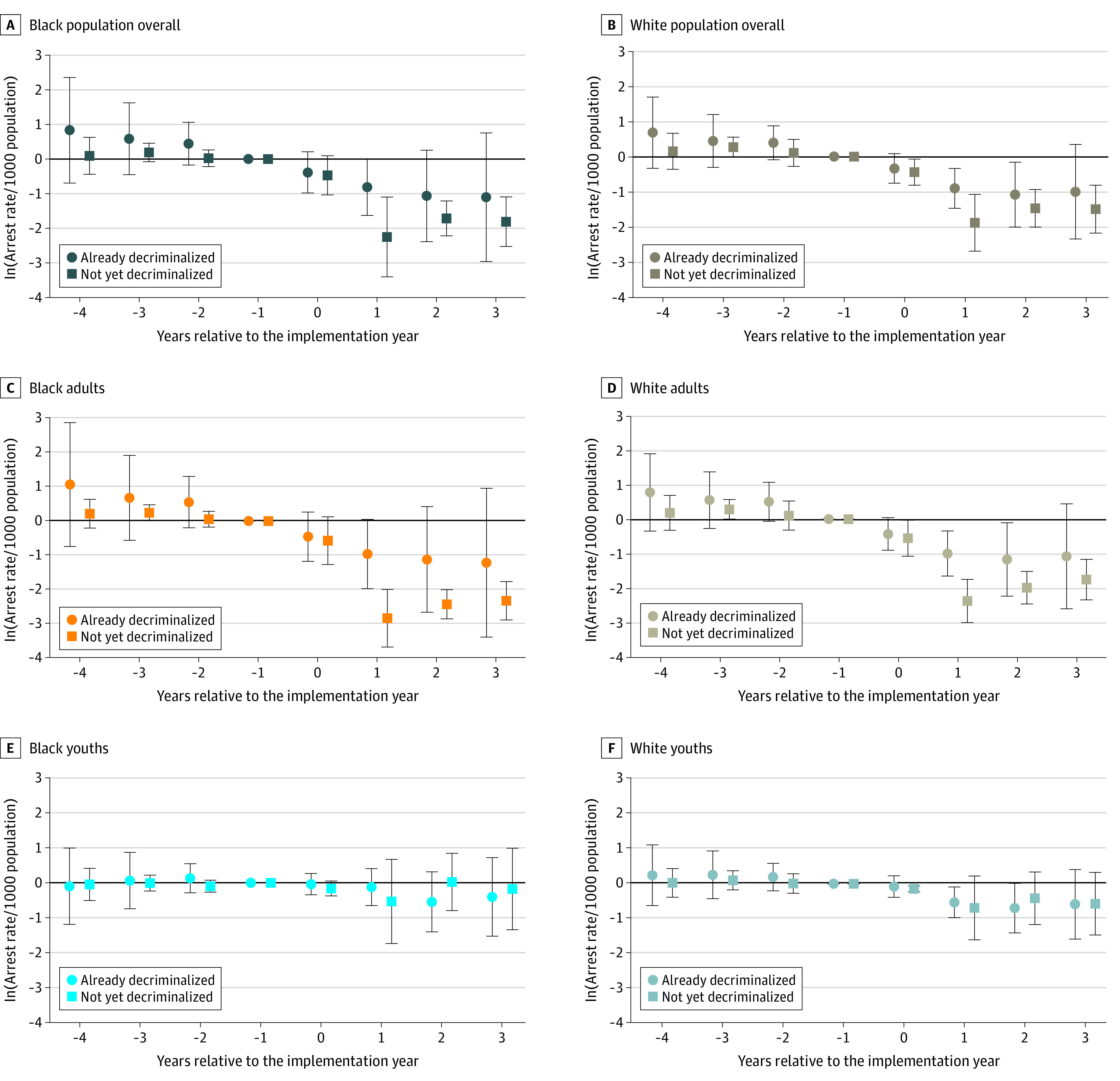
Event Study by Racial Group, With Arrest Rates per 1000 Population per Year Estimated coefficients are shown for the overall population, adults, and youths by racial group; error bars indicate 95% CIs. The year before implementation was the reference (omitted) year. The estimated coefficient should be interpreted as relative to this year. The final lag or lead points accumulated all years beyond (ie, −4 included year −4 and earlier; 3 included year 3 and later). All regressions also included controls for the presence of medical cannabis legalization, share of the population with less than a high school diploma or equivalent, share of female individuals in the population, share of individuals in the population from racial and ethnic minority groups, share of youths in the population, number of police officers per 1000 population, unemployment rate, income per capita in 2019 thousand dollars, poverty rate, state and year indicators, and state-specific time trends. Standard errors were clustered at the state level. All regressions were weighted by state population averaged over the study period (2010-2019).

Figure 1 and Figure 2 report results from the event study. It appeared that the estimated associations were not driven by differences in prelegalization trends between RCL and non-RCL states. The validity of the difference-in-differences design was hence supported. The association might be dynamic: in most cases, the decline seemed to be larger in magnitude after the year of RCL implementation. The dynamic association should be interpreted with caution, however, because some states had very limited time points in the post-RCL period.

eFigures 4 and 5 in Supplement 1 report results from the leave-one-out analysis. Overall, it seems that the main findings were not driven by a specific RCL state.

## Discussion

This cross-sectional study examined the association of RCL with changes in cannabis possession arrests and tested whether the association differed between RCL states with and without decriminalization already in place. Our results suggest that RCL was associated with a substantial decrease in adult arrest rates in both types of RCL states for the study period (2010-2019). For adults, the magnitude of the decrease (40.0%-76.3%) associated with RCL was comparable to that associated with cannabis decriminalization.^3,4,5^ States that had already decriminalized cannabis before RCL saw a smaller magnitude of decline (40.0%) than states that had not decriminalized cannabis before RCL (76.3%). These findings suggest that implementing RCL may be associated with a further reduction in adult arrest rates even after a state decriminalizes cannabis.

Consistent with a 2019 study by Plunk et al,^5^ we did not find an association between RCL and cannabis possession arrests among youths regardless of decriminalization status in RCL states. This finding was not surprising because RCL intends to legalize cannabis use among adults but not youths. If youth arrest is more concerning because of the prolonged, adverse health and socioeconomic consequences from adolescence to adulthood,^22^ cannabis decriminalization may be a preferred strategy because it removes criminal penalties not only for adults but also youths and also reduces arrests in both age groups.^3,4,5^

Despite similar rates of cannabis use, Black individuals are reportedly 3 to 4 times more likely to be arrested for cannabis possession compared with White individuals.^10^ A previous study estimated that cannabis decriminalization was associated with an approximately 17.0% reduction in racial disparities in arrests among Black and White adults.^3^ Our results suggest that RCL might not provide additional benefits in terms of reducing racial disparities compared with decriminalization. Nonetheless, we should note that the decrease in cannabis possession arrests after RCL was substantial for both Black and White adults, demonstrating an overall change in law enforcement behaviors.

If we compare the benefits of RCL and cannabis decriminalization based solely on their associations with cannabis possession arrests, this study and the existing literature suggest that both RCL and decriminalization are associated with a sizable reduction in adult arrest rates.^3,4,5^ Even after decriminalization was implemented, adults could still benefit from a further reduction in arrests under RCL. The argument that RCL could reduce individual contact with the criminal justice system is supported. Nonetheless, decriminalization has additional benefits; for example, it was also associated with reductions in arrests among youths and in racial disparities among Black and White individuals.^3^ That said, the choice of RCL and decriminalization approaches should be made with a holistic evaluation of all benefits and costs. The effect on the criminal justice system is a major consideration but should not be the only one. Other considerations could include effects on public health,^6^ the economy, and society. Policy makers are encouraged to adopt a strategy only when the total benefits outweigh the total costs.

### Limitations

This cross-sectional study is not without limitations. First, police agency reporting to the UCRP is voluntary. To account for measurement errors, we followed previous studies to weight the regressions by population.^19,20^ However, this approach may not have fully eliminated the bias. Second, cannabis possession arrests may be underestimated due to the hierarchical reporting in the UCRP. We believe that it should not bias the difference-in-differences estimates unless RCL implementation was associated with changes in the reporting method or the volume of severe crimes. Third, UCRP information on race may be inaccurate. Fourth, we were not able to examine other races or ethnicities due to data limitations. Fifth, time-varying unobserved confounding factors may not have been fully accounted for by the difference-in-differences design. Furthermore, findings from the 9 RCL states may not necessarily generalize to other US states or outside of the US setting. Finally, it may take time for the effects on law enforcement behaviors to fully materialize after RCL. The post-RCL period in this study might be too short to capture changes in youths and racial disparities. A re-examination with a longer post-RCL period is warranted.

## Conclusions

The findings of this repeated cross-sectional study suggest that RCL was associated with a substantial decrease in adult arrests in US states that had already decriminalized cannabis, albeit of a smaller magnitude compared with RCL states that had not. RCL did not appear to be associated with changes in arrest rates among youths or disparities in arrest rates among Black and White individuals.

## Notes

Supplement 1. **eTable 1.** States Included and Excluded in the Study **eFigure 1.** Trends in Overall Cannabis Possession Arrest Rates in Recreational Cannabis Legalization States **eFigure 2.** Trends in Adult Cannabis Possession Arrest Rates in Recreational Cannabis Legalization States **eFigure 3.** Trends in Youth Cannabis Possession Arrest Rates in Recreational Cannabis Legalization States **eTable 2.** Regression Results for the Overall Population: Recreational Cannabis Legalization States Without Cannabis Decriminalization Already in Place **eTable 3.** Regression Results for Adults: Recreational Cannabis Legalization States Without Cannabis Decriminalization Already in Place **eTable 4.** Regression Results for Youths: Recreational Cannabis Legalization States Without Cannabis Decriminalization Already in Place **eTable 5.** Regression Results for the Overall Population: Recreational Cannabis Legalization States With Cannabis Decriminalization Already in Place **eTable 6.** Regression Results for Adults: Recreational Cannabis Legalization States With Cannabis Decriminalization Already in Place **eTable 7.** Regression Results for Youths: Recreational Cannabis Legalization States With Cannabis Decriminalization Already in Place **eFigure 4.** Leave-One-Out Analysis for Recreational Cannabis Legalization States Without Decriminalization Already in Place **eFigure 5.** Leave-One-Out Analysis for Recreational Cannabis Legalization States With Decriminalization Already in Place

Supplement 2.
Data Sharing Statement
