# Supplementary material for: Association of Recreational Cannabis Legalization With Cannabis Possession Arrest Rates in the US
Source: JAMA Netw Open. 2022 Dec 5;5(12):e2244922. doi: 10.1001/jamanetworkopen.2022.44922 (PMC9855298; doi:10.1001/jamanetworkopen.2022.44922)
Supplement: Supplement 1. — eTable 1. States Included and Excluded in the Study eFigure 1. Trends in Overall Cannabis Possession Arrest Rates in Recreational Cannabis Legalization States eFigure 2. Trends in Adult Cannabis Possession Arrest Rates in Recreational Cannabis Legalization States eFigure 3. Trends in Youth Cannabis Possession Arrest Rates in Recreational Cannabis Legalization States eTable 2. Regression Results for the Overall Population: Recreational Cannabis Legalization States Without Cannabis Decriminalization Already in Place eTable 3. Regression Results for Adults: Recreational Cannabis Legalization States Without Cannabis Decriminalization Already in Place eTable 4. Regression Results for Youths: Recreational Cannabis Legalization States Without Cannabis Decriminalization Already in Place eTable 5. Regression Results for the Overall Population: Recreational Cannabis Legalization States With Cannabis Decriminalization Already in Place eTable 6. Regression Results for Adults: Recreational Cannabis Legalization States With Cannabis Decriminalization Already in Place eTable 7. Regression Results for Youths: Recreational Cannabis Legalization States With Cannabis Decriminalization Already in Place eFigure 4. Leave-One-Out Analysis for Recreational Cannabis Legalization States Without Decriminalization Already in Place eFigure 5. Leave-One-Out Analysis for Recreational Cannabis Legalization States With Decriminalization Already in Place [file jamanetwopen-e2244922-s001.pdf]

Supplementary Online Content

Gunadi C, Shi Y. Association of recreational cannabis legalization with cannabis possession arrest rates in the US. *JAMA Netw Open*. 2022;5(12):e2244922. doi:10.1001/jamanetworkopen.2022.44922

eTable 1. States Included and Excluded in the Study

eFigure 1. Trends in Overall Cannabis Possession Arrest Rates in Recreational Cannabis Legalization States

eFigure 2. Trends in Adult Cannabis Possession Arrest Rates in Recreational Cannabis Legalization States

eFigure 3. Trends in Youth Cannabis Possession Arrest Rates in Recreational Cannabis Legalization States

eTable 2. Regression Results for the Overall Population: Recreational Cannabis Legalization States Without Cannabis Decriminalization Already in Place

eTable 3. Regression Results for Adults: Recreational Cannabis Legalization States Without Cannabis Decriminalization Already in Place

eTable 4. Regression Results for Youths: Recreational Cannabis Legalization States Without Cannabis Decriminalization Already in Place

eTable 5. Regression Results for the Overall Population: Recreational Cannabis Legalization States With Cannabis Decriminalization Already in Place

eTable 6. Regression Results for Adults: Recreational Cannabis Legalization States With Cannabis Decriminalization Already in Place

eTable 7. Regression Results for Youths: Recreational Cannabis Legalization States With Cannabis Decriminalization Already in Place

eFigure 4. Leave-One-Out Analysis for Recreational Cannabis Legalization States Without Decriminalization Already in Place

eFigure 5. Leave-One-Out Analysis for Recreational Cannabis Legalization States With Decriminalization Already in Place

This supplementary material has been provided by the authors to give readers additional information about their work.

© 2022 Gunadi C et al. *JAMA Network Open*.

eTable 1. States Included and Excluded in the Study

	States (Effective Year of RCL)	Sources
**Types of States Included in the Study**		
RCL States Without Decriminalization Already in Place (4 States)		Abouk et al. (2021), Midgette and Reuter (2020), Grucza et al. (2018), Plunk et al. (2019), Pacula et al. (2003)
	Washington (2012)	
	Alaska (2015)	
	Nevada (2017)	
	Michigan (2018)	
RCL States With Decriminalization Already in Place (5 States)		
	Oregon (2015) - Decriminalized in 1973	
	California[1] (2016) - Decriminalized in 2011	
	Massachusetts (2016) - Decriminalized in 2009	
	Maine (2017) - Decriminalized in 1975	
	Vermont[1] (2018) - Decriminalized in 2013	
Non-RCL States (22 States)	Alabama, Arizona, Georgia, Hawaii, Idaho, Indiana, Iowa, Minnesota, Mississippi, New Jersey, North Carolina, Oklahoma, Pennsylvania, South Carolina, South Dakota, Tennessee, Texas, Utah, Virginia, West Virginia, Wisconsin, Wyoming	
**Types of States Excluded from the Study[2,3]**		
Non-RCL States that Decriminalized Cannabis in the Study Period (8 States)	Connecticut, Rhode Island, Maryland, Delaware, Illinois, New Hampshire, New Mexico, North Dakota	Grucza et al. (2018), Plunk et al. (2019), MPP (2020)
Non-RCL States that Changed Penalties for Cannabis Possession in the Study Period (9 States)	Arkansas, Nebraska, Kansas, Kentucky, Louisiana, New York, Missouri, Ohio, Montana	
Notes: a. Because California and Vermont decriminalized cannabis during the study period, we excluded state*year observations in years before and when California and Vermont implemented cannabis decriminalization. b. Florida was excluded because it did not report arrest statistics to UCR for most years in the study period. c. Colorado was also excluded because it had reporting errors in UCR in the study period.		

© 2022 Gunadi C et al. *JAMA Network Open*.

eFigure 1. Trends in Overall Cannabis Possession Arrest Rates in Recreational Cannabis Legalization States

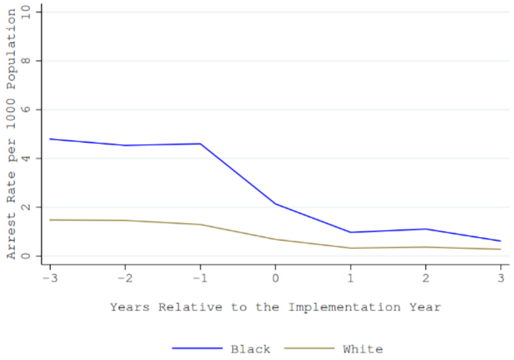

(a) Not Yet Decriminalized

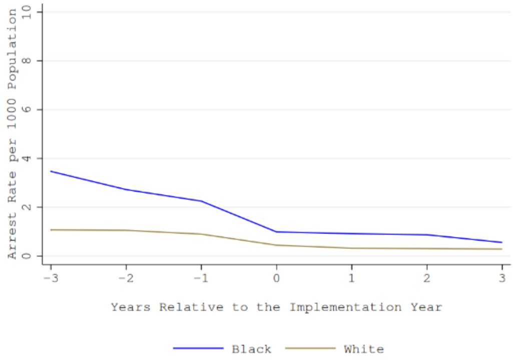

(b) Already Decriminalized

Notes:
Event time 0 was the implementation year of RCL, -1 was the year before the implementation year, and 1 was the year after the implementation year. The rest of the event time can be interpreted similarly.

© 2022 Gunadi C et al. *JAMA Network Open*.

eFigure 2. Trends in Adult Cannabis Possession Arrest Rates in Recreational Cannabis Legalization States

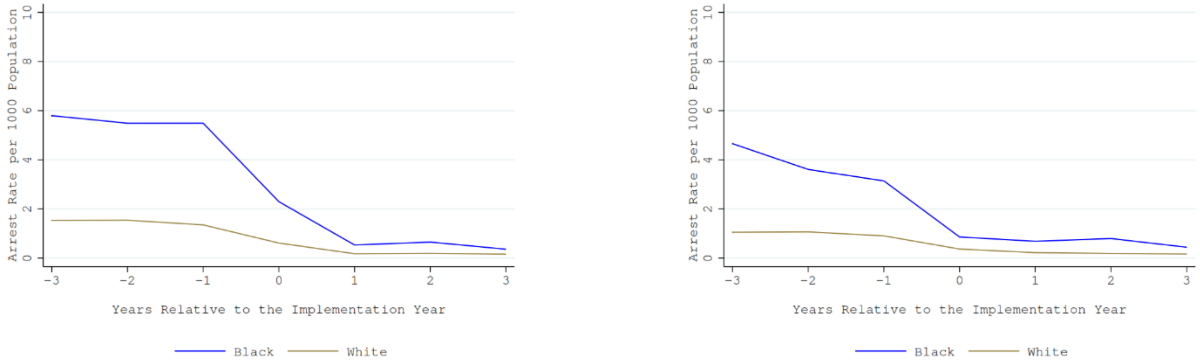

(a) Not Yet Decriminalized

(b) Already Decriminalized

Notes:
Event time 0 was the implementation year of RCL, -1 was the year before the implementation year, and 1 was the year after the implementation year. The rest of the event time can be interpreted similarly.

© 2022 Gunadi C et al. *JAMA Network Open*.

eFigure 3. Trends in Youth Cannabis Possession Arrest Rates in Recreational Cannabis Legalization States

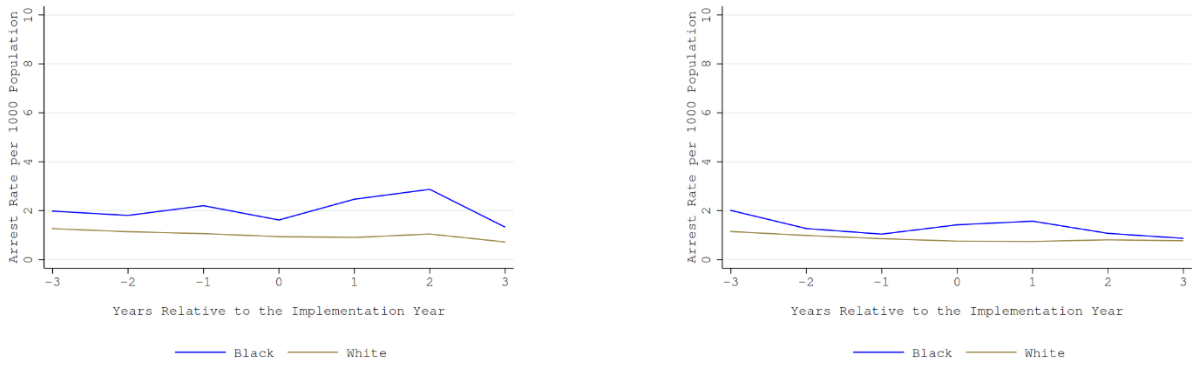

(a) Not Yet Decriminalized

(b) Already Decriminalized

Notes:
Event time 0 was the implementation year of RCL, -1 was the year before the implementation year, and 1 was the year after the implementation year. The rest of the event time can be interpreted similarly.

© 2022 Gunadi C et al. *JAMA Network Open*.

eTable 2. Regression Results for the Overall Population: Recreational Cannabis Legalization States Without Cannabis Decriminalization Already in Place

	ln(Cannabis Possession Arrest Rate per 1,000 Population)		
	**All Races**	**Black**	**White**
	**Coefficient (standard error) [95% Confidence Interval] P-Value**		
RCL	-1.16	-1.21	-1.09
	(0.12)	(0.15)	(0.10)
	[-1.41 -0.91]	[-1.52 -0.89]	[-1.30 -0.88]
	<0.001	<0.001	<0.001
Medical Cannabis Legalization	-0.21	-0.27	-0.22
	(0.14)	(0.14)	(0.13)
	[-0.50 0.08]	[-0.55 0.0077]	[-0.49 0.048]
	0.15	0.06	0.10
Share of Population (< High School Diploma/Equivalent)	11.70	17.66	20.84
	(23.69)	(19.55)	(19.48)
	[-37.091 60.50]	[-22.61 57.92]	[-19.28 60.97]
	0.63	0.38	0.30
Share of Females in the Population	-6.0052	-31.79	0.96
	(41.82)	(36.83)	(37.02)
	[-92.14 80.13]	[-107.65 44.069]	[-75.29 77.21]
	0.89	0.40	0.98
Share of Non-White in the Population	-2.34	-5.48	-0.85
	(3.90)	(3.25)	(3.87)
	[-10.38 5.70]	[-12.17 1.21]	[-8.83 7.12]
	0.55	0.10	0.83
Share of Youths (<18) in the Population	-14.24	-18.37	-29.98
	(27.75)	(24.63)	(28.13)
	[-71.40 42.92]	[-69.10 32.35]	[-87.91 27.95]
	0.61	0.46	0.30
Police Officers per 1,000	0.041	0.026	0.029
	(0.11)	(0.13)	(0.11)

© 2022 Gunadi C et al. *JAMA Network Open*.

	[-0.19 0.28]	[-0.25 0.30]	[-0.20 0.26]
	0.72	0.85	0.80
Unemployment Rate	-16.53	-19.95	-18.75
	(13.82)	(14.03)	(11.56)
	[-44.997 11.95]	[-48.84 8.93]	[-42.55 5.06]
	0.24	0.17	0.12
Income per Capita (in Thousands)	0.11	0.11	0.11
	(0.077)	(0.076)	(0.071)
	[-0.050 0.27]	[-0.049 0.27]	[-0.03 0.26]
	0.17	0.17	0.12
Poverty Rate	18.49	16.12	19.55
	(11.70)	(10.68)	(11.55)
	[-5.61 42.59]	[-5.87 38.11]	[-4.23 43.33]
	0.13	0.14	0.10

Notes:

a. All regressions also included controls for the presence of medical cannabis legalization, the share of the population with less than high school diploma or equivalent, the share of females in the population, the share of non-Whites in the population, the share of youths in the population, the number of police officers per 1,000 population, unemployment rate, income per capita in 2019 thousand dollars, poverty rate, state and year indicators, and state-specific time trends.

b. Standard errors were clustered at state level.

c. All regressions were weighted by state population averaged over the 2010-2019 period.

© 2022 Gunadi C et al. *JAMA Network Open*.

eTable 3. Regression Results for Adults: Recreational Cannabis Legalization States Without Cannabis Decriminalization Already in Place

	ln(Cannabis Possession Arrest Rate per 1,000 Population)		
	**All Races**	**Black**	**White**
	**Coefficient (standard error) [95% Confidence Interval] P-Value**		
RCL	-1.44	-1.51	-1.37
	(0.12)	(0.16)	(0.092)
	[-1.67 -1.20]	[-1.85 -1.18]	[-1.56 -1.18]
	<0.001	<0.001	<0.001
Medical Cannabis Legalization	-0.22	-0.27	-0.23
	(0.15)	(0.15)	(0.14)
	[-0.53 0.084]	[-0.57 0.033]	[-0.52 0.048]
	0.15	0.08	0.10
Share of Population (< High School Diploma/Equivalent)	8.51	15.32	19.25
	(24.60)	(20.31)	(20.31)
	[-42.16 59.17]	[-26.51 57.15]	[-22.59 61.091]
	0.73	0.46	0.35
Share of Females in the Population	-0.54	-23.60	6.52
	(44.60)	(40.068)	(39.78)
	[-92.38 91.31]	[-106.12 58.92]	[-75.41 88.45]
	0.99	0.56	0.87
Share of Non-White in the Population	-1.71	-4.97	-0.54
	(4.21)	(3.60)	(4.19)
	[-10.38 6.97]	[-12.39 2.44]	[-9.17 8.09]
	0.69	0.18	0.90
Share of Youths (<18) in the Population	-16.94	-22.35	-34.26
	(31.27)	(28.91)	(31.45)

© 2022 Gunadi C et al. *JAMA Network Open*.

	[-81.34 47.45]	[-81.90 37.19]	[-99.04 30.52]
	0.59	0.45	0.29
Police Officers per 1,000	0.045	0.065	0.027
	(0.12)	(0.13)	(0.11)
	[-0.19 0.28]	[-0.21 0.34]	[-0.21 0.26]
	0.70	0.63	0.82
Unemployment Rate	-17.39	-19.36	-20.17
	(14.35)	(14.20)	(12.03)
	[-46.95 12.17]	[-48.60 9.88]	[-44.94 4.59]
	0.24	0.19	0.11
Income per Capita (in Thousands)	0.11	0.12	0.11
	(0.085)	(0.091)	(0.077)
	[-0.065 0.28]	[-0.067 0.31]	[-0.047 0.27]
	0.21	0.20	0.16
Poverty Rate	18.43	15.85	19.51
	(11.91)	(11.09)	(11.79)
	[-6.11 42.96]	[-6.99 38.69]	[-4.78 43.79]
	0.13	0.17	0.11

Notes:

a. All regressions also included controls for the presence of medical cannabis legalization, the share of the population with less than high school diploma or equivalent, the share of females in the population, the share of non-Whites in the population, the share of youths in the population, the number of police officers per 1,000 population, unemployment rate, income per capita in 2019 thousand dollars, poverty rate, state and year indicators, and state-specific time trends.
b. Standard errors were clustered at state level.
c. All regressions were weighted by state population averaged over the 2010-2019 period.

© 2022 Gunadi C et al. *JAMA Network Open*.

eTable 4. Regression Results for Youths: Recreational Cannabis Legalization States Without Cannabis Decriminalization Already in Place

	ln(Cannabis Possession Arrest Rate per 1,000 Population)		
	**All Races**	**Black**	**White**
	**Coefficient (standard error) [95% Confidence Interval] P-Value**		
RCL	-0.38	-0.24	-0.36
	(0.22)	(0.27)	(0.20)
	[-0.85 0.078]	[-0.81 0.32]	[-0.77 0.047]
	0.10	0.38	0.08
Medical Cannabis Legalization	-0.18	-0.30	-0.15
	(0.090)	(0.093)	(0.086)
	[-0.37 0.0030]	[-0.49 -0.10]	[-0.33 0.024]
	0.05	0.004	0.09
Share of Population (< High School Diploma/Equivalent)	16.54	18.42	12.37
	(19.19)	(14.92)	(15.16)
	[-22.98 56.06]	[-12.30 49.14]	[-18.86 43.59]
	0.40	0.23	0.42
Share of Females in the Population	-18.59	-28.26	-14.31
	(25.30)	(21.99)	(23.46)
	[-70.69 33.51]	[-73.55 17.03]	[-62.63 34.0026]
	0.47	0.21	0.55
Share of Non-White in the Population	-5.81	-5.86	-3.16
	(3.00075)	(3.20)	(2.94)
	[-11.99 0.37]	[-12.46 0.74]	[-9.22 2.89]
	0.06	0.08	0.29
Share of Youths (<18) in the Population	-3.98	-29.52	-6.58
	(19.038)	(35.72)	(18.11)
	[-43.19 35.23]	[-103.08 44.04]	[-43.87 30.71]
	0.84	0.42	0.72

© 2022 Gunadi C et al. *JAMA Network Open*.

Police Officers per 1,000	-0.020	-0.060	0.018
	(0.14)	(0.18)	(0.12)
	[-0.31 0.27]	[-0.42 0.30]	[-0.22 0.26]
	0.89	0.74	0.88
Unemployment Rate	-12.47	-16.08	-11.35
	(8.99)	(8.48)	(8.12)
	[-30.98 6.04]	[-33.55 1.39]	[-28.08 5.37]
	0.18	0.07	0.17
Income per Capita (in Thousands)	0.10	0.079	0.10
	(0.076)	(0.072)	(0.073)
	[-0.06 0.25]	[-0.07 0.23]	[-0.05 0.25]
	0.22	0.28	0.20
Poverty Rate	12.33	12.49	11.15
	(8.46)	(7.70)	(7.78)
	[-5.10 29.76]	[-3.36 28.34]	[-4.87 27.17]
	0.16	0.12	0.16

Notes:

a. All regressions also included controls for the presence of medical cannabis legalization, the share of the population with less than high school diploma or equivalent, the share of females in the population, the share of non-Whites in the population, the share of youths in the population, the number of police officers per 1,000 population, unemployment rate, income per capita in 2019 thousand dollars, poverty rate, state and year indicators, and state-specific time trends.

b. Standard errors were clustered at state level.

c. All regressions were weighted by state population averaged over the 2010-2019 period.

© 2022 Gunadi C et al. *JAMA Network Open*.

eTable 5. Regression Results for the Overall Population: Recreational Cannabis Legalization States With Cannabis Decriminalization Already in Place

	ln(Cannabis Possession Arrest Rate per 1,000 Population)		
	**All Races**	**Black**	**White**
	**Coefficient (standard error) [95% Confidence Interval] P-Value**		
RCL	-0.40	-0.37	-0.41
	(0.14)	(0.14)	(0.13)
	[-0.68 -0.12]	[-0.66 -0.085]	[-0.67 -0.15]
	0.007	0.01	0.003
Medical Cannabis Legalization	-0.084	-0.078	-0.12
	(0.062)	(0.061)	(0.068)
	[-0.21 0.043]	[-0.20 0.047]	[-0.26 0.021]
	0.19	0.21	0.09
Share of Population (< High School Diploma/Equivalent)	19.19	16.54	27.45
	(21.16)	(18.77)	(18.87)
	[-24.29 62.68]	[-22.05 55.13]	[-11.34 66.23]
	0.37	0.39	0.16
Share of Females in the Population	-12.52	-40.39	-4.88
	(45.76)	(41.51)	(40.077)
	[-106.58 81.54]	[-125.71 44.94]	[-87.26 77.50]
	0.79	0.34	0.90
Share of Non-White in the Population	-2.75	-3.96	-1.51
	(3.29)	(2.19)	(3.65)
	[-9.51 4.02]	[-8.46 0.53]	[-9.00 5.99]
	0.41	0.08	0.68
Share of Youths (<18) in the Population	7.79	10.76	-9.62
	(17.08)	(17.13)	(19.93)

© 2022 Gunadi C et al. *JAMA Network Open*.

	[-27.33 42.91]	[-24.45 45.98]	[-50.60 31.35]
	0.65	0.54	0.63
Police Officers per 1,000	0.10*	0.15	0.083
	(0.042)	(0.047)	(0.043)
	[0.014 0.19]	[0.056 0.25]	[-0.0064 0.17]
	0.03	0.003	0.07
Unemployment Rate	-1.79	1.51	-5.22
	(5.27)	(3.97)	(4.31)
	[-12.62 9.04]	[-6.65 9.66]	[-14.08 3.65]
	0.74	0.71	0.24
Income per Capita (in Thousands)	0.057	0.088	0.065
	(0.10)	(0.10)	(0.087)
	[-0.14 0.26]	[-0.11 0.28]	[-0.11 0.24]
	0.57	0.37	0.46
Poverty Rate	16.43	13.29	18.24
	(12.068)	(10.84)	(11.96)
	[-8.37 41.24]	[-8.98 35.57]	[-6.33 42.82]
	0.19	0.23	0.14

Notes:

a. All regressions also included controls for the presence of medical cannabis legalization, the share of the population with less than high school diploma or equivalent, the share of females in the population, the share of non-Whites in the population, the share of youths in the population, the number of police officers per 1,000 population, unemployment rate, income per capita in 2019 thousand dollars, poverty rate, state and year indicators, and state-specific time trends.
b. Standard errors were clustered at state level.
c. All regressions were weighted by state population averaged over the 2010-2019 period.

© 2022 Gunadi C et al. *JAMA Network Open*.

eTable 6. Regression Results for Adults: Recreational Cannabis Legalization States With Cannabis Decriminalization Already in Place

	ln(Cannabis Possession Arrest Rate per 1,000 Population)		
	All Races	Black	White
	Coefficient (standard error) [95% Confidence Interval] P-Value		
RCL	-0.51	-0.43	-0.53
	(0.14)	(0.18)	(0.13)
	[-0.80 -0.22]	[-0.81 -0.06]	[-0.80 -0.27]
	0.001	0.03	<0.001
Medical Cannabis Legalization	-0.081	-0.055	-0.12
	(0.062)	(0.061)	(0.072)
	[-0.21 0.046]	[-0.18 0.071]	[-0.27 0.027]
	0.20	0.38	0.10
Share of Population (< High School Diploma/Equivalent)	17.84	16.28	27.37
	(21.28)	(19.30)	(19.18)
	[-25.91 61.59]	[-23.38 55.94]	[-12.06 66.80]
	0.41	0.41	0.17
Share of Females in the Population	-13.024	-32.53	-4.45
	(48.22)	(45.35)	(42.50)
	[-112.15 86.10]	[-125.74 60.69]	[-91.82 82.91]
	0.79	0.48	0.92
Share of Non-White in the Population	-4.67	-7.31	-3.25
	(3.90)	(3.47)	(4.0060)
	[-12.68 3.34]	[-14.44 -0.19]	[-11.48 4.99]
	0.24	0.05	0.43

© 2022 Gunadi C et al. *JAMA Network Open*.

Share of Youths (<18) in the Population	27.16	42.62	4.19
	(18.52)	(22.23)	(20.88)
	[-10.91 65.24]	[-3.08 88.32]	[-38.74 47.12]
	0.16	0.07	0.84
Police Officers per 1,000	0.11	0.16	0.079
	(0.063)	(0.057)	(0.060)
	[-0.02 0.24]	[0.04 0.28]	[-0.04 0.20]
	0.11	0.009	0.20
Unemployment Rate	0.15	5.50	-4.035
	(5.32)	(4.9992)	(4.33)
	[-10.78 11.08]	[-4.78 15.77]	[-12.94 4.87]
	0.98	0.28	0.36
Income per Capita (in Thousands)	0.068	0.069	0.072
	(0.10)	(0.12)	(0.090)
	[-0.14 0.28]	[-0.17 0.31]	[-0.11 0.26]
	0.51	0.55	0.43
Poverty Rate	17.26	13.97	19.19
	(12.29)	(11.27)	(12.22)
	[-8.0047 42.53]	[-9.19 37.13]	[-5.93 44.31]
	0.17	0.23	0.13

Notes:

a. All regressions also included controls for the presence of medical cannabis legalization, the share of the population with less than high school diploma or equivalent, the share of females in the population, the share of non-Whites in the population, the share of youths in the population, the number of police officers per 1,000 population, unemployment rate, income per capita in 2019 thousand dollars, poverty rate, state and year indicators, and state-specific time trends.
b. Standard errors were clustered at state level.
c. All regressions were weighted by state population averaged over the 2010-2019 period.

© 2022 Gunadi C et al. *JAMA Network Open*.

eTable 7. Regression Results for Youths: Recreational Cannabis Legalization States With Cannabis Decriminalization Already in Place

	ln(Cannabis Possession Arrest Rate per 1,000 Population)		
	**All Races**	**Black**	**White**
	**Coefficient (standard error) [95% Confidence Interval] P-Value**		
RCL	-0.19	-0.10	-0.18
	(0.10)	(0.065)	(0.093)
	[-0.39 0.021]	[-0.23 0.034]	[-0.37 0.013]
	0.08	0.14	0.07
Medical Cannabis Legalization	-0.074	-0.10	-0.092
	(0.10)	(0.17)	(0.067)
	[-0.28 0.13]	[-0.45 0.25]	[-0.23 0.045]
	0.46	0.55	0.18
Share of Population (< High School Diploma/Equivalent)	21.95	15.31	17.65
	(17.68)	(15.02)	(14.58)
	[-14.38 58.29]	[-15.56 46.17]	[-12.32 47.63]
	0.23	0.32	0.24
Share of Females in the Population	-12.59	-27.20	-9.01
	(27.98)	(26.72)	(25.45)
	[-70.10 44.92]	[-82.12 27.72]	[-61.32 43.30]
	0.66	0.32	0.73
Share of Non-White in the Population	-4.10	0.32	-2.27
	(2.03)	(3.33)	(2.55)
	[-8.26 0.07]	[-6.52 7.16]	[-7.50 2.96]
	0.05	0.93	0.38
Share of Youths (<18) in the Population	-26.80	-63.49	-24.72
	(22.84)	(38.65)	(20.59)
	[-73.76 20.16]	[-142.94 15.97]	[-67.05 17.60]

© 2022 Gunadi C et al. *JAMA Network Open*.

	0.25	0.11	0.24
Police Officers per 1,000	0.046	0.057	0.066
	(0.084)	(0.12)	(0.068)
	[-0.13 0.22]	[-0.18 0.30]	[-0.074 0.21]
	0.59	0.63	0.34
Unemployment Rate	-6.97	-7.10	-6.94
	(5.39)	(6.34)	(5.04)
	[-18.041 4.10]	[-20.13 5.92]	[-17.29 3.41]
	0.21	0.27	0.18
Income per Capita (in Thousands)	0.013	0.023	0.023
	(0.076)	(0.068)	(0.071)
	[-0.14 0.17]	[-0.12 0.16]	[-0.12 0.17]
	0.87	0.74	0.74
Poverty Rate	9.21	8.11	8.92
	(8.54)	(7.75)	(7.77)
	[-8.34 26.77]	[-7.83 24.053]	[-7.06 24.90]
	0.29	0.31	0.26

Notes:

a. All regressions also included controls for the presence of medical cannabis legalization, the share of the population with less than high school diploma or equivalent, the share of females in the population, the share of non-Whites in the population, the share of youths in the population, the number of police officers per 1,000 population, unemployment rate, income per capita in 2019 thousand dollars, poverty rate, state and year indicators, and state-specific time trends.
b. Standard errors were clustered at state level.
c. All regressions were weighted by state population averaged over the 2010-2019 period.

© 2022 Gunadi C et al. *JAMA Network Open*.

eFigure 4. Leave-One-Out Analysis for Recreational Cannabis Legalization States Without Decriminalization Already in Place

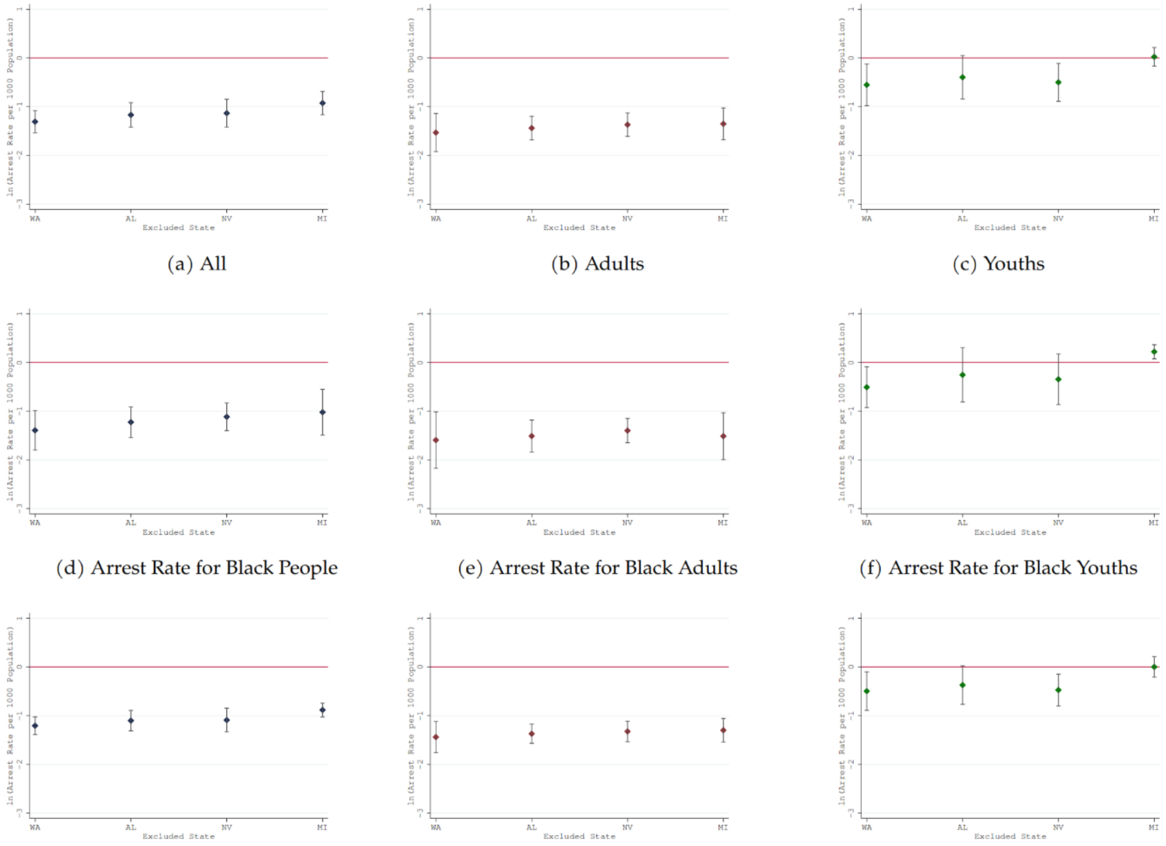

(a) All (b) Adults (c) Youths

(d) Arrest Rate for Black People (e) Arrest Rate for Black Adults (f) Arrest Rate for Black Youths

(g) Arrest Rate for White People (h) Arrest Rate for White Adults (i) Arrest Rate for White Youths

Notes:

a. Estimated coefficient and 95% confidence intervals are reported.
b. All regressions also included controls for the presence of medical cannabis legalization, the share of the population with less than high school diploma or equivalent, the share of females in the population, the share of non-Whites in the population, the share of youths in the population, the number of police officers per 1,000 population, unemployment rate, income per capita in 2019 thousand dollars, poverty rate, state and year indicators, and state-specific time trends.
c. Standard errors were clustered at state level.
d. All regressions were weighted by state population averaged over the 2010-2019 period.

© 2022 Gunadi C et al. *JAMA Network Open*.

eFigure 5. Leave-One-Out Analysis for Recreational Cannabis Legalization States With Decriminalization Already in Place

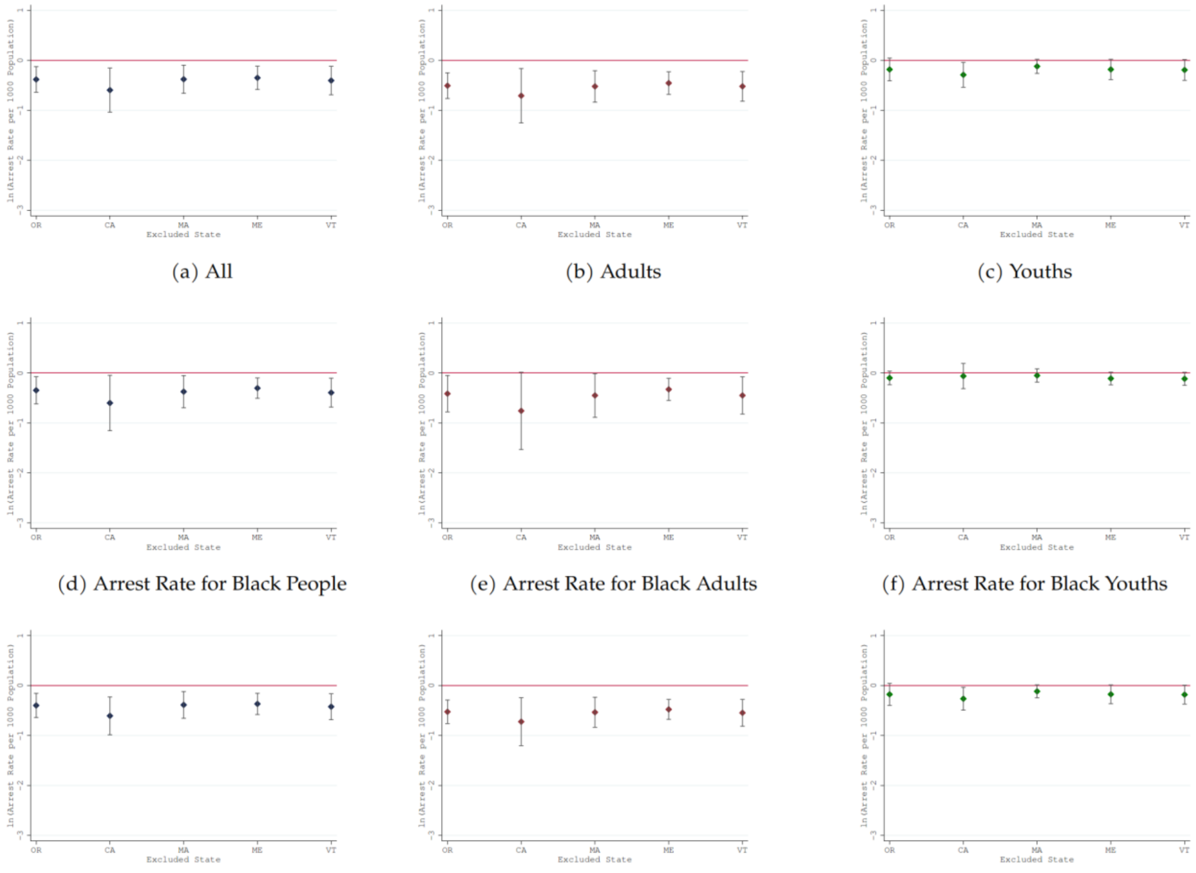

(a) All (b) Adults (c) Youths

(d) Arrest Rate for Black People (e) Arrest Rate for Black Adults (f) Arrest Rate for Black Youths

(g) Arrest Rate for White People (h) Arrest Rate for White Adults (i) Arrest Rate for White Youths

Notes:

a. Estimated coefficient and 95% confidence intervals are reported.
b. All regressions also included controls for the presence of medical cannabis legalization, the share of the population with less than high school diploma or equivalent, the share of females in the population, the share of non-Whites in the population, the share of youths in the population, the number of police officers per 1,000 population, unemployment rate, income per capita in 2019 thousand dollars, poverty rate, state and year indicators, and state-specific time trends.
c. Standard errors were clustered at state level.
d. All regressions were weighted by state population averaged over the 2010-2019 period.

© 2022 Gunadi C et al. *JAMA Network Open*.
