# Supplementary material for: Association of Recreational Cannabis Legalization With Cannabis Possession Arrest Rates in the US
Source: JAMA Netw Open. 2022 Dec 5;5(12):e2244922. doi: 10.1001/jamanetworkopen.2022.44922 (PMC9855298; doi:10.1001/jamanetworkopen.2022.44922)
Supplement: Supplement 2. — Data Sharing Statement [file jamanetwopen-e2244922-s002.pdf]

Data Sharing Statement

Gunadi. Association of Recreational Cannabis Legalization With Cannabis Possession Arrest Rates in the US. *JAMA Netw Open*. Published December 05, 2022. doi:10.1001/jamanetworkopen.2022.44922

Data
Data available: No

Additional Information
Explanation for why data not available: Our study used publicly available secondary data.
